# Molecular mechanisms of EBV-driven cell cycle progression and oncogenesis

**DOI:** 10.1007/s00430-018-0570-1

**Published:** 2018-11-01

**Authors:** Huali Yin, Jiani Qu, Qiu Peng, Runliang Gan

**Affiliations:** 1 Medical School, Cancer Research Institute, Key Laboratory of Tumor Cellular and Molecular Pathology of Hunan Province, University of South China, Chang Sheng Xi Avenue 28, Hengyang, 421001 Hunan People’s Republic of China; 2 Department of Pathology, Central Hospital of Shaoyang, Hunan, China

**Keywords:** Epstein–Barr virus (EBV), Neoplasm, Cell cycle, Molecular mechanism

## Abstract

The early stage of oncogenesis is linked to the disorder of the cell cycle. Abnormal gene expression often leads to cell cycle disorders, resulting in malignant transformation of human cells. Epstein–Barr virus (EBV) is associated with a diverse range of human neoplasms, such as malignant lymphoma, nasopharyngeal carcinoma and gastric cancer. EBV mainly infects human lymphocytes and oropharyngeal epithelial cells. EBV is latent in lymphocytes for a long period of time, is detached from the cytoplasm by circular DNA, and can integrate into the chromosome of cells. EBV expresses a variety of latent genes during latent infection. The interaction between EBV latent genes and oncogenes leads to host cell cycle disturbances, including the promotion of G_1_/S phase transition and inhibition of cell apoptosis, thereby promoting the development of EBV-associated neoplasms. Molecular mechanisms of EBV-driven cell cycle progression and oncogenesis involve diverse genes and signal pathways. Here, we review the molecular mechanisms of EBV-driven cell cycle progression and promoting oncogenesis.

## Introduction

Uncontrolled cell proliferation is a hallmark of cancer, with abnormal genes expressed in cancer cells directly involved in regulating cell cycle. The fundamental task of the cell cycle is to make sure that DNA is faithfully replicated once during S phase and that identical chromosome copies are distributed equally to two daughter cells during M phase. The decision of cell mitosis occurs as cells pass a restriction point (R point) late in *G*_1_, after which they enter S phase. There is a precise mechanism of cell cycle regulation in a normal cell. Malfunctions in cell cycle give access to cells to gain uncontrolled growth characteristics, primarily hyperproliferation and a low rate of apoptosis, ultimately leading to oncogenesis [1]. The presence of the corresponding growth factor or proliferation signal allows cells to enter S phase from *G*_0_/*G*_1_ phase through the restriction point, completing the entire cell cycle.

**Fig. 1:**
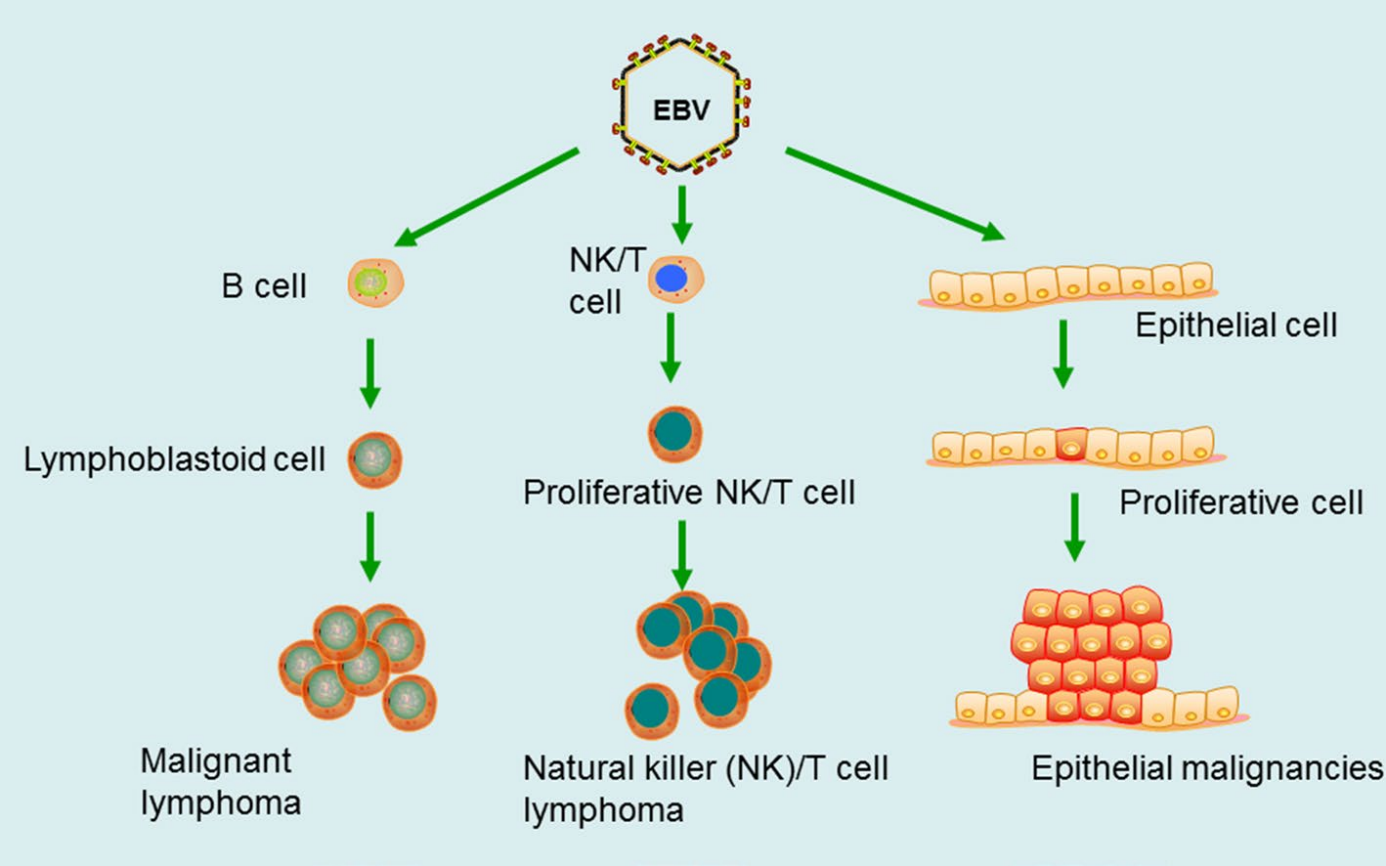
EBV is associated with a diverse range of human neoplasms. EBV mainly infects B lymphocytes, making them malignant transformation, and then forms a malignant lymphoma, such as BL. EBV may transform epithelial cells into epithelial malignancies, such as NPC, EBV-GC. Recent studies have found that EBV can also infect NK/T cells to form a natural killer/T cell lymphoma (NK/T cell lymphoma)

It is more than 50 years since Epstein–Barr virus (EBV), the first human oncogenic virus, was discovered [2]. EBV, with the most common and persistent infection in humans, and roughly 95% of the world’s populations sustaining an asymptomatic life-long infection, mainly infects lymphocytes and oropharyngeal epithelial cells. EBV has subsequently been found to be associated with a diverse range of neoplasms, such as Burkitt lymphoma (BL) [3], Hodgkin lymphoma (HL) [4], AIDs-related non-Hodgkin lymphoma [5], post-transplant lymphoproliferative disorders (PTLD) [6], diffuse large B cell lymphoma (DLBCL) [7], NK/T cell lymphoma [8], nasopharyngeal carcinoma (NPC) [9], and EBV-positive gastric cancer (EBV-GC) [10] (Fig. 1). EBV can transform human B lymphocytes *in vitro* and make them immortalized [11, 12]. Human peripheral blood lymphocytes were transplanted to severe combined immunodeficient (SCID) mice, and EBV-associated human-derived lymphomas generated in hu-PBL/SCID chimeric mice [13, 14]. This EBV-induced lymphoma model is similar to AIDs-associated lymphoma and post-transplant lymphoproliferative disease (PTLD).

EBV virion, its diameter is about 150–170 nm, is composed of lipoprotein capsules and icosahedral nucleocapsid that include 162 shell particles. The viral genome consists of about 170 kb double-stranded DNA. EBV long-term latent in lymphocytes in the form of circular DNA free in the cytoplasm can be integrated into cell chromosome. The EBV genome within LCLs usually exists in multiple copies of extrachromosomal circular genetic materials known as episomes and expresses all latent genes (refer to as latency III or the ‘growth programme’), including six Epstein–Barr virus nuclear antigens [EBNA 1, 2, 3A, 3B and 3C and EBNA leader protein (EBNA‑LP)], latent membrane proteins LMP-1 and LMP-2 (which encodes two isoforms, LMP-2A and LMP-2B), EBV-encoded small RNAs (EBER1 and EBER2) and microRNAs (miRNAs). During different stages of B cell differentiation *in vivo*, EBV expresses either the latency III programme, or one of two alternative forms of virus latency (known as latency I and latency II) (Table 1). EBV-associated B cell lymphomas express three latent types (I, II, or III), which type of latency depends on the B cell stage of tumor origin. The majority of EBV-positive Burkitt lymphomas (BL) is characterized by latency I, but some BL cell lines drift towards latency III during culture in vitro, such as Raji cells [15, 16]. In contrast, EBV infection does not induce clonal expansion in primary epithelial cells, all EBV-associated epithelial cancers express a latency II programme [17]. This viral gene expression pattern is essential for driving resting B cells into the cell cycle and maintains a proliferative state.

**Table 1:** Different latent states of Epstein–Barr virus in EBV-associated neoplasms

EBV latent type	EBV latent genes	Diseases
I	EBNA-1, EBERs	BL
II	EBNA-1, LMP-1, LMP-2A, LMP-2B, EBERs, BARTs	NPC, HL, EBV-GC, NK/T cell lymphomas
III	EBNA-1, LMP-1, LMP-2A, LMP-2B, EBNA-2, EBNA-3A/B/C/LP, EBERs, EBV-miR-BHRF1/BARTs	AIDs-related NHL, partial BL (after extensive passage in vitro), PTLD, DLBCL

## Latent membrane protein 1

Latent membrane protein 1 (LMP-1) is considered to be an oncogenic protein whose signal region contains three sites: carboxy-terminal activating region 1 (CTAR1) (194–232 a.a.), CTAR2 (351–386 a.a.) and CTAR3 (275–330 a.a.) that directly interact with some cell factors and activate NF-κB, JNK (c-Jun N-terminal kinase), p38 MAPK, JAK/STAT, and PI3K/Akt signal pathways which involved in cell cycle progression [18]. There is a difference between the LMP-1 gene derived from nasopharyngeal carcinoma and derived from B95-8 cells. Chinese nasopharyngeal carcinoma, primarily carry deletion type LMP-1 with a 30-bp deletion (Cao LMP1), which has stronger transformation ability [19]. Sueur et al. found that weak IFN-γ expression and specific alteration of the cell cycle might be a way for del30-LMP1 infected cells to escape the immune anti-viral response and to promote the development of cancer [20]. Studies have characterized the ability of LMP-1 to recruit TRAFs (TRAF1, -2, -3, and -5) to CTAR1 through a consensus TRAF-binding site at positions 204 to 208. Xie’s data revealed that CD40 and LMP1 unexpectedly used TRAF3 in different ways and that TRAF3 was required for LMP1-mediated activation of B cells [21]. LMP1-CTAR1 is necessary for rodent fibroblast transformation. LMP1-CTAR1 has the unique ability to induce expression of EGFR and TRAF1, and can deregulate molecules involved in G_1_/S cell cycle progression, such as an inhibitor of differentiation or DNA binding 1 (Id1), the CDK inhibitor p27^Kip1^, CDK2, and Rb [22]. Increased levels of hyperphosphorylated CDK2 and total Rb, cellular markers involved in G_1_/S cell cycle progression, were observed in LMP-1, LMP1Δ204–208, 1–220, and 1–231. LMP-1 regulates telomerase activity through the p16^INK4A^/Rb/E2F1, PI3K-AKT and JNK signaling pathways to promote cell immortalization [23].

CTAR2 is known to engage the JNK and NF-κB pathways. Elipoulos et al. reported that induction of LMP-1 directly activated functional JNK [24]. Then Wan et al. showed that CTAR2 specifically recruits TRAF6 in the LMP1-mediated JNK pathway [25]. Kutz et al. demonstrated that inhibition of the JNK pathway by the JNK-specific inhibitor SP600125 resulted in reduced tumor growth of LCLs in SCID mice. These indicated that the LMP1-induced JNK pathway was required for lymphoblasts to progress efficiently through the cell cycle and was used to maintain expression of the c-Jun and G_2_/M cell cycle kinase Cdc2 [26]. JNK activation mediated by LMP-1 was responsible for upregulation of CCL3 and CCL4 required for LCL survival and growth [27]. CD40, a member of the tumor necrosis factor (TNF) receptor family, plays an essential role in T cell-dependent immune responses. Hömig-Hölzel et al. found that B cell-specific expression of LMP1/CD40 activated the MAPKs/JNK/ERK and the noncanonical NF-κB pathway [28]. The concerted action of these signaling pathways ultimately leads to B cell lymphomagenesis. The DOK1 gene is a newly identified tumor suppressor gene with altered expression via hypermethylation of its promoter in a variety of human cancers. Siouda et al. found that LMP-1 down-regulated DOK1 expression by altering the composition of the E2F transcription complex [29]. Lo et al. identified a novel function of LMP1 to inhibit the LKB1-AMPK pathway through phosphorylation of LKB1 at serine 428 with subsequent suppression of the phosphorylation of AMPK and its substrates, ACC and Raptor, which finally promoted proliferation and transformation of human nasopharyngeal epithelial cells [30]. Xiao et al. demonstrated that upregulation of HK2 by LMP-1 conferred NPC cells with a proliferative advantage and the ability to resist apoptosis [31]. Comprehensively, LMP-1 interacts with cell cycle-related molecules such as NF-κB, JNK, STAT, PI3K, Akt, p27^kip^, CDK2, and Rb, thereby, promoting G_1_/S phase transition, conferring cells proliferation advantages, and anti-apoptosis ability.

## Latent membrane protein 2A

Latent membrane protein 2A (LMP-2A) of EB virus is expressed during different latency stages of EBV-infected B cells. Mancao et al. demonstrated that LMP-2A could rescue BCR-GC B cells from apoptosis in an *in vivo* situation. This indicated that EBV involved in the initial steps of lymphomagenesis of GC-derived B cell lymphomas directly [32]. Wasil et al. demonstrated that LMP-1 and LMP-2A proteins jointly contributed to oncogenic mechanisms by modulating DNA repair [33]. LMP-2A contains 12 transmembrane domains and both the N and C termini face the cytosol. The cytoplasmic amino-terminal domain of LMP-2A contains an immunoreceptor tyrosine activation motif (ITAM). ITAM in the LMP-2A N terminus is constitutively phosphorylated and activates the Syk protein tyrosine kinase (PTK). Fukuda et al. found that the interaction of the LMP-2A ITAM with Syk was a key step for LMP-2A mediated transformation [34]. Engels et al. discovered that ITAM in the LMP-2A N terminus induced a ligation-independent activation signal during its initial expression which mimics that of the antigen-activated BCR, and thus passing survival signals to B cells, which is an important pathway for EBV regulation of cell growth [35]. LMP-2A promotes cell transformation and survival through the activity of host cell signaling pathways. LMP2A-induced migration activity correlates with the ITAM/Syk signaling [36]. Fukuda et al. found that ITAM in the LMP-2A N terminus was required for LMP2A-mediated Akt phosphorylation and anchorage-independent cell growth in several human cell lines [34]. LMP-2A dramatically affects epithelial cell transformation mediated through activation of the PI3-kinase–Akt pathway [37, 38]. Swart et al. reported that LMP-2A promotes constitutive phosphorylation of Akt through the PI3-K pathway [39], and subsequent studies have found that PI3-K/mTOR inhibitor, NVP-BEZ235, is effective against follicular lymphoma [40]. LMP-2A couples with MYC to promote G_1_/S transition and hyperproliferation of B lymphocytes through promoting p27^kip1^ degradation at the early stage of lymphomagenesis [41]. Fish et al. demonstrated that LMP-2A promoted hyperproliferation of B cells by way of up-regulating MYC expression and MYC-dependent degradation of the tumor suppressor p27^kip1^ [42]. Incrocci et al. found that LMP-2A enhanced IL-10 production through the activation of Bruton’s tyrosine kinase and STAT3, then B cell survival [43]. Wang et al. found that knockdown of LMP-2A inhibited the proliferation and clonogenicity of GT38 cells which were arrested in the G_0_/G_1_ phase [44]. As was stated above, LMP-2A promotes cell transformation by interacting with PI3K/Akt and ITAM/Syk, and LMP-2A can promote excessive proliferation of B cells by up-regulating MYC and degrading p27^kip^.

## Epstein–Barr virus nuclear antigen 1

Epstein–Barr virus nuclear antigen 1 (EBNA-1) is an essential viral protein, expressed in virus latency as well as EBV-associated neoplasm [45]. The EBV origin of plasmid replication (oriP), which is a 1.7 kb size area on the EBV chromosome, contains two functional elements: DS element and FR element [46]. FR, consisting of 20 binding sites for EBNA-1, is of great importance for viral replication and also works as a transcriptional enhancer. Malik-Soni et al. demonstrated that the histone chaperone nucleophosmin was recruited by EBNA1 to the FR element, which was required for EBNA1-mediated transcriptional activation [47]. EBNA-1 binds to the FR element as a transcriptional activator to activate the expression of the promoter of the viral Cp and LMP genes, then enhances the expression of LMP-1, consequently promotes cell proliferation [48]. Boreström et al. discovered that the cell cycle regulatory protein E2F1, the E2F-binding protein ARID3A, and the B-cell-specific transcription factor Oct-2 bind the core promoter sequence of the EBV Cp as well as the minimal FR sequence containing eight EBNA1-binding sites, which was necessary for transcriptional activation [49]. EBNA-1 together with a large cohort of cellular genes resorts to the survival and proliferation functions. EBNA-1 depletion from latently infected LCLs results in the loss of cell proliferation and the loss of gene expression for some EBNA1-bound genes, including MEF2B, EBF1 and IL 6R. These findings suggest that EBNA-1, as a critical regulator of transcription of host cell genes, has a vital importance for enhancing survival of latently infected cells [50].

## Epstein–Barr virus nuclear antigen 2 and its co-activator EBV nuclear antigen leader protein

Epstein–Barr virus nuclear antigen 2 (EBNA-2) is initially expressed after the infection of EBV, which is absolutely necessary for virus-mediated transformation. Cyclin D2 and CDK4 are both elements of the basic cell cycle machinery and driving cell cycle progression in early G_1_. The cooperation between EBNA-2 and EBNA-LP (leader protein) can induce cyclin D2 expression in resting B cells. LMP-1 and c-Myc are directly activated by EBNA-2, indicating that activation of c-Myc by EBNA-2 is an important step in the process of EBV-induced proliferation and immortalization [51]. EBNA-2 is able to expedite cellular proliferation and survival of EBV-infected B cells, which most likely by way of acting as a transcriptional activator of cellular and viral gene expression. It is a functional homolog of activated Notch receptor. Konforte et al. discovered that complete JAK/STAT pathway was indispensable to the IL-21-mediated regulation of EBNA-2 and LMP-1 protein expression [52]. These suggest that EBNA-2 plays an important role in cell cycle and cell proliferation.

Epstein–Barr nuclear antigen leader protein (EBNA-LP) is a phosphoprotein [53]. Phosphorylation appears to occur predominantly on serine residues, and while this can be detected throughout the cell cycle, it is hyperphosphorylated during G_2_/M and hypophosphorylated during G_1_/S phase [54]. Kato et al. found that cellular protein kinase cdc2 targets the functional phosphorylation site Ser-35 of EBNA-LP in vitro, which promoted G_1_/S transition [55]. A strong co-activation between EBNA-LP and EBNA-2 was reported by Tierney et al. [56]. Co-expression of EBNA-2 and EBNA-LP in primary B cells induces the expression of cyclin D2, thus promoting the G_1_/S transition. Szymula et al. found that EBNA-LP did not simply co-operate with EBNA2 in activating gene transcription, but rather facilitates the recruitment of several transcription factors to the viral genome, to enable transcription of virus latency genes. And they also found that EBNA-LP was essential for the survival of EBV-infected naïve B cells [57]. These findings imply that EBNA-2 plays an important role in cell cycle and cell proliferation. Cooperation between EBNA-2 and EBNA-LP can induce cyclin D2 expression in resting B cells.

## Epstein–Barr virus nuclear antigen 3A

Epstein–Barr virus nuclear antigen 3A (EBNA-3A) has been shown to play a role in the regulation of cell survival in B cells immortalized by EBV [58]. Tursiella et al. discovered that knockdown of EBNA-3A expression resulted in abrupt cell cycle arrest in G_0_/G_1_ phase that was concomitant with the conversion of retinoblastoma protein (Rb) to its hypophosphorylated state, followed by a loss of Rb protein; They also found that p21^WAF1/CIP1^ expression was elevated following RNAi-mediated knockdown of EBNA-3A in LCLs [59]. Myc-interacting zinc finger protein-1 (MIZ-1) is a transcription factor initially characterized as a binding partner of MYC. MIZ-1 activates the transcription of a number of target genes including the cell cycle inhibitor CDKN2B. Bazot et al. reported that EBNA-3A protein inhibited CDKN2B transcription via interaction with MIZ-1, thus promoting the cell proliferation [60]. Carboxyl-terminal binding protein (CtBP) has been shown to be a highly conserved co-repressor of transcription that is important in development, cell cycle regulation, and transformation. EBNA-3A can physically and functionally interact with CtBP, whose interaction has been shown to depend on two cryptic sites located near the COOH terminus of the protein that binds CtBP synergistically. These sites appear to be necessary for EBNA3A-mediated repression of transcription and, as with EBNA-3C, binding to CtBP correlated with the ability of EBNA-3A to co-operate with oncogenic Ras in primary rodent fibroblasts. Skalska et al. showed that EBNA-3A, EBNA-3C, and CtBP were all involved in the epigenetic repression of p16^INK4A^ expression that is necessary for the proliferation of EBV-transformed B cells [61].

## Epstein–Barr virus nuclear antigen 3C

Epstein–Barr virus nuclear antigen 3C (EBNA-3C) is a small subset of latent antigens critical for the transformation of human primary B lymphocytes into continuously proliferating lymphoblastoid cell lines (LCLs) *in vitro* through manipulation of a number of major cellular pathways. Moreover, EBNA-3C can stabilize c-Myc and enhance c-Myc-dependent transcription. EBNA-3C residues 130 to 190 recruit and modulate the activity of retinoblastoma (Rb) and p27, both major regulators of the mammalian cell cycle. The inclusion of c-Myc in the group of cellular targets modulated by this domain further accentuates the importance of these critical residues of EBNA-3C in bypassing the cell cycle checkpoints [62]. An EBV recombinant deleted for residues 130–159 in EBNA-3C can deregulate p53/Mdm2 and cyclin D1/CDK6 which results in apoptosis and reduce cell proliferation [63]. The inhibitor of growth (ING), a tumor suppressor, can be divided into three categories: one is ING1 and ING2, and ING4 and ING5 are classified as type II, while ING3 is different from other members [64, 65]. Saha et al. found that EBNA-3C nullified the positive regulation of both ING4 and ING5 in the tumor suppressive activity of p53 [66]. The p73 protein has structural and functional homology with the tumor suppressor p53. Sahu et al. discovered that the repressive effects of EBNA-3C on p73 function increased the efficiency of EBV-mediated lymphomagenesis. Interestingly, there is a colocalization between EBNA-3C and nuclear p73 [67]. The role of the pRb-E2F pathway in the regulation of cell cycle progression, particularly the G_1_/S transition, is well established. E2F1 plays a dual role in controlling cell growth and apoptosis. For example, elevated expression of E2F1 promotes cell cycle progression by driving quiescent cells into S phase [68]. However, E2F1 expression can also induce apoptosis in the absence of proliferative signals [69]. Saha et al. discovered that EBNA-3C efficiently blocked E2F1-mediated apoptosis, as well as its anti-proliferative effects in a p53-independent manner, in response to DNA damage [70]. E2F6 is one of the E2F family members with a unique property of transcriptional repression. E2F6 recruited together with EBNA3C binds E2F1 promoter and inhibits its activity, which contributes to B cell proliferation by reducing the expression of E2F1 [71]. EBNA-3C and EBNA-3A jointed repression of CDKN2A p16^INK4A^ and p14^ARF^ was essential for LCL growth, was reported by Maruo et al. [72]. These suggest that EBNA-3C can facilitate G_1_ to S transition. Accordingly, EBNA-3C inhibits p73, p14^ARF^ and p16^INK4A^ to promote G_1_/S transition. At the same time, the combination of EBNA-3C and E2F6 can effectively block E2F1-mediated apoptosis and promote cell proliferation.

Additionally, Rovedo et al. [73] showed that LMP-2B negatively regulates the activity of LMP-2A. As described by White et al. [74], EBNA-3B is dispensable for B cell transformation in vitro. EBNA-3B is a virally encoded tumor suppressor gene that inhibits EBV-transformed B cell proliferation to ensure long-term survival of the persistently infected host.

## EBV-encoded small RNAs (EBER1 and EBER2)

EBV-encoded small RNAs (EBERs, specifically EBER-1 and EBER-2) are transcribed by RNA polymerase III into non-translated RNAs of 167 and 172 nucleotides, respectively, and form stem-loop structures by intramolecular base-pairing, giving rise to double-stranded RNA like structures. EBERs are considered as reliable markers for in situ hybridization to detect EBV infection in clinical samples of gastric carcinoma, lymphoma, nasopharyngeal carcinoma and etc. [75–78]. Herbert et al. found that EBER1 and EBER2 were functional back-ups of viral oncoprotein LMP-1, which activated the oncogenic PI3K/Akt signaling pathway [79]. Komano et al. demonstrated that EBER-expressing Akata cell clones restored the malignant phenotype, resistance to apoptosis, and up-regulated expression of a Bcl-2 protein to levels comparable to the restoration rates for EBER expression in EBV-reinfected cell clones [80]. Etodolac, cyclooxygenase-2 (COX-2) inhibitor, induced apoptosis via a COX-2 independent pathway. Kobayashi et al. demonstrated that the expressions of EBER-1 and EBER-2 in EBV-positive Daudi and Raji cells were reduced, resulting in down-regulation of Bcl-2 by treatment with etodolac. This result indicated etodolac inhibits EBERs expression and induced apoptosis via a Bcl-2-regulated pathway [81]. EBERs inhibit the activity of the double-stranded RNA-dependent protein kinase, PKR, which is reputed to act as a tumor suppressor [82]. On the other hand, EBER can up-regulate IL-6 expression and activate signal transducers and activators of transcription (STAT), thus inhibiting the expression of cell cycle inhibition gene p21 and p27 and releasing the inhibition of CDK2 and CDK4, promoting the G_1_/S transition finally [83]. In short, EBER promotes G_1_/S transition by activating the oncogenic PI3K/Akt signaling pathway and inhibiting the tumor suppressor PKR and the cell cycle inhibitors p21 and p27.

## EBV-encoded microRNAs (EBV miRNAs)

**Fig. 2:**
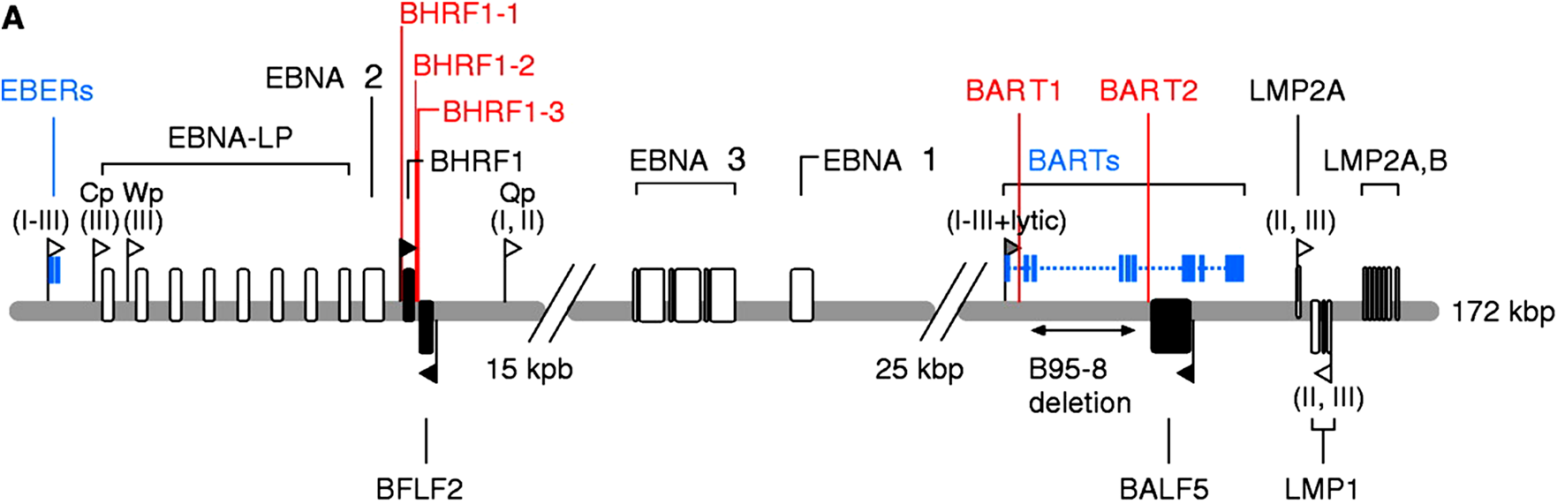
Genomic positions of EBV-encoded miRNAs. EBV can encode approximately 23 precursors and 44 mature miRNAs. EBV-encoded miRNAs are grouped into two clusters: BHRF1 and BART clusters [84]

EBV has been recently found to encode microRNAs (miRNAs), which expressed in infected B cells and some EBV-associated neoplasms. EBV can encode approximately 23 precursors and 44 mature miRNAs. EBV miRNAs are grouped into two clusters located either adjacent to the BHRF1 gene or in introns contained within the viral BART transcripts (Fig. 2) [84]. The first miRNA cluster is located within the mRNA of the BHRF1 (Bam HI fragment H rightward open reading frame 1) gene encoding a distant Bcl-2 homolog (miR-BHRF1-1 to miR-BHRF1-3) [85]. MiR-BHRF1-1 is located in the 5′UTR (untranslated region) and miR-BHRF1-2 and -3 are positioned in the 3′UTR of the BHRF1 mRNA [84]. To understand the function of the BHRF1 miRNA cluster, Feederle et al. constructed a virus mutant that lacks all its three members (∆123) and a revertant virus, and showed that B cell transforming capacity of the ∆123 EBV mutant was reduced by more than 20-fold, comparative to wild-type or revertant viruses. It displayed slower growth in B cells which infected the knock-out virus, that exhibited a twofold reduction in the percentage of cells entering the cell cycle S phase [86]. PRDM1 (PR domain zinc finger protein 1, also known as BLIMP-1) is a tumor suppressor gene, Ma et al. found that EBV-miR-BHRF1-2 inhibition up-regulated PRDM1 protein expression in lymphoblastoid cell lines (LCL), which was important for EBV-transformed B cell proliferation [87].

EBV BamHI‑A rightward transcript (BART) miRNAs have nearly 30 kinds of BART miRNAs, which overexpressed in EBV-associated malignancies. Lung et al. found that down-modulation of LMP-2A expression by miR-BART22 might permit the escape of EBV-infected cells from host immune surveillance, which may facilitate NPC carcinogenesis [88]. Lei et al. reported that EBV-encoded miR-BART3* miRNA targets DICE1 tumor suppressor to promote cellular growth and transformation in NPC [89]. Kang et al. demonstrated that EBV miR-BART miRNAs (miR-BART3, 6, 8, 16 and 22) expressed in EBV-infected epithelial tumor cell line AGS show anti-apoptotic activity to promote epithelial cell survival [90]. Lu et al. found that miR-BART6-3p inhibited the EBV-triggered IFN-β response and facilitated EBV infection through targeting the 3′ UTR of RIG-I mRNA [91]. Wong et al. discovered that EBV microRNAs deregulated the canonical Wnt signaling pathway, which down-regulated Wnt inhibitory genes such as Wnt inhibitory factor 1 (WIF1), MAP kinase (MAPK)-related NEMO-like kinase (NLK) and adenomatous polyposis coli (APC), thus promoting oncogenesis [92]. Zhao et al. found that the activity of the Wnt pathway in EBV-associated tumors might be enhanced by miR-BART19-3p [93]. Zhou et al. discovered that cellular miRNA (miR-142) that functions together with EBV-BART-6-3p as oncogenes to suppressed the expression of PTEN (Phosphatase and tensin homolog) which is a known tumor suppressor [94]. Vereide et al. found that EBV BART miRNAs were able to promote B cell proliferation at early stage of EBV infection, and could target caspase3 and inhibit cell apoptosis, increasing the number of cells entering S phase [95]. Qiu et al. found that the BART miRNAs potentiate tumor growth and development *in vivo* [96]. Hooykaas et al. identified that miR-BART16 abrogated the production of IFN-stimulated genes in response to IFN-α stimulation and inhibited the antiproliferative effect of IFN-α on latently infected BL cells, which promoted proliferation [97]. Lung et al. found that the four EBV miRNAs, BART5-5p, BART7-3p, BART9-3p and BART14-3p, worked cooperatively to modulate ATM activity in response to DNA damage and to maintain viral latency, contributing to the tumorigenesis of NPC [98].

These findings indicate that EBV-encoded microRNAs play a contributing role in EBV-associated malignancies. EBV-miR-BHRF1-2 interacts with the tumor suppressor gene PRDM1 and plays an important role in cell proliferation. EBV-miR-BARTs can escape immune surveillance by down-regulating LMP-2A, and promote cell proliferation by down-regulating Wnt’s inhibitory gene and tumor suppressor PTEN.

## Conclusions

**Fig. 3:**
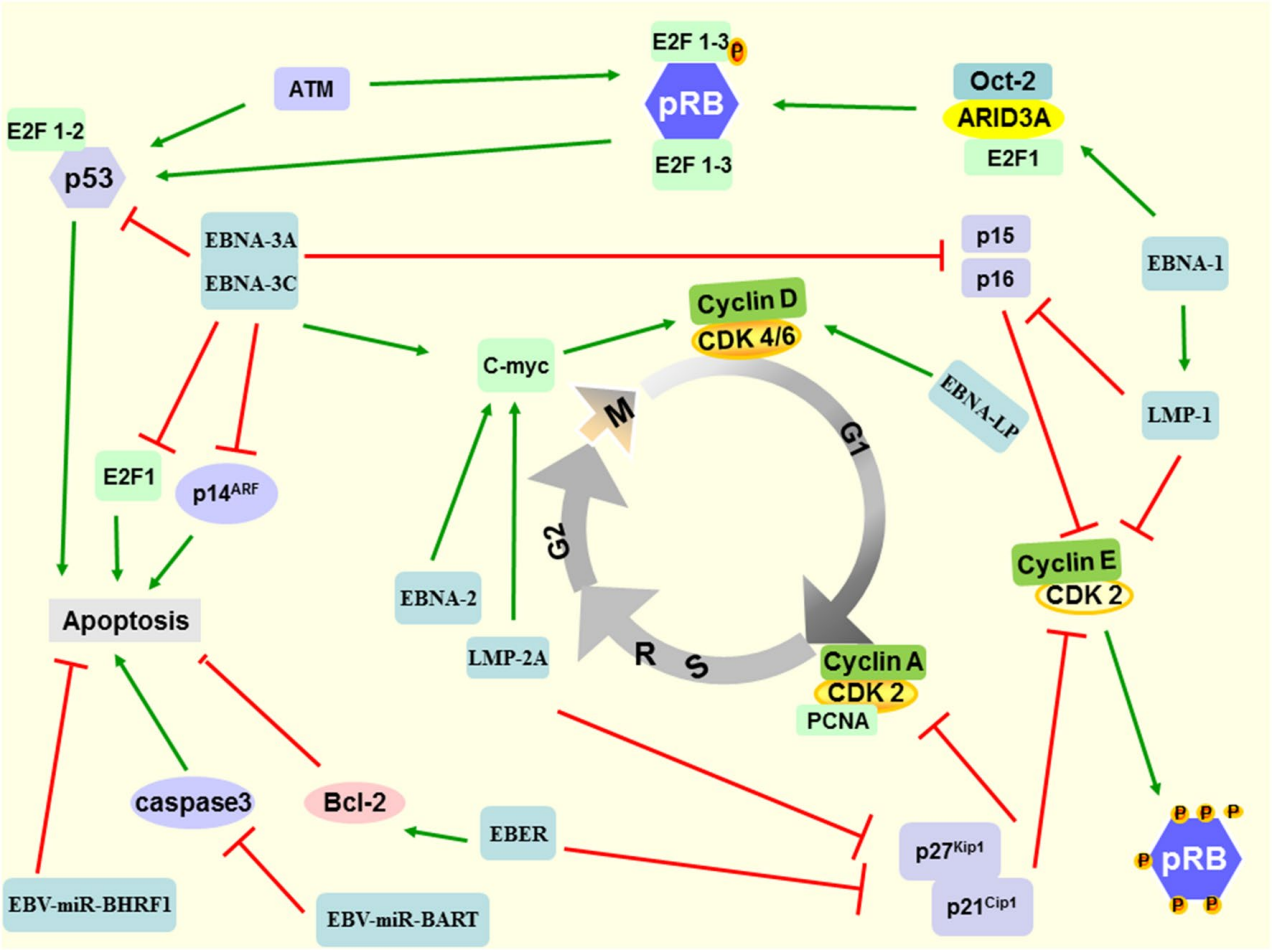
A schematic diagram of EB virus involved in the G_1_/S transition. Epstein–Barr virus infection is an early event in the development of malignancies. The latent proteins and miRNAs encoded by EBV in host cells alone or in combination drive the cell cycle through a variety of pathways. LMP-1 regulates telomerase activity through the p16^INK4A^/Rb/E2F1 signaling pathway to promote cell immortalization. LMP-2A couples with c-Myc to promote G_1_/S transition and hyperproliferation of B lymphocytes through promoting the expression of cyclin D and the degradation of p27^kip1^ at the early stage of oncogenesis. The cell cycle regulatory protein E2F1, the E2F-binding protein ARID3A, and the B-cell-specific transcription factor Oct-2 bind EBNA-1, which are necessary for transcriptional activation. EBNA-1 also enhances expression of LMP-1, and then promotes cell proliferation. The interaction between EBNA-2, EBNA-3C, and c-Myc further activates cyclin D2 and CDK4, then promoting the cell from G1 phase into S phase. EBNA-3A and EBNA-3C down-regulate the expression of p15^INK4b^, p16^INK4a^, and p14^ARF^, thereby inhibiting apoptosis. EBAN-3C can directly bind to p53, to a certain extent, inhibit its transcriptional activity. EBERs can up-regulate Bcl-2 and down-regulate p21^cip1^ and p27^kip1^, thereby releasing the inhibition of CDK4 and CDK2 and promoting the cell cycle from G_1_ phase to S phase. EBV-miR-BHRF1 inhibits apoptosis in B lymphocytes and epithelial cells. EBV-miR-BARTs can target caspase 3, thereby inhibiting apoptosis and increasing the number of cells entering the S phase

As mentioned above, EBV plays a key role in driving cell cycle and oncogenesis of EBV-positive neoplasms. Multiple genes and signal pathways are involved in the occurrence of EBV-related neoplasms, including the interaction of virus gene and host genes (Fig. 3). Epstein–Barr virus genes activate oncogenes such as Bcl-2 and MYC, as well as signaling pathways such as NF-κB, JNK, JAK/STAT, and PI3K/Akt, and inhibit tumor suppressor DOK1, PKR, p53, PRDM1, DICE1, PTEN, and p27^kip1^, p21^WAF1/CIP1^, p16^INK4A^, p73, etc. The time from viral infection to tumorigenesis is usually shorter in EBV-associated neoplasms with specific oncogene activation. EBV-encoded genes rapidly lead to oncogenesis by activating cellular oncogenes or interacting with proteins in host cells. The latent proteins and miRNAs encoded by EB virus in host cells alone or in combination drive the cell cycle through a variety of pathways.

Identifying the molecular mechanism of EBV-driven cell cycle progression and oncogenesis may help to diagnose and guide clinical medication. Nevertheless, the precise mechanisms still remain unclear, especially those related to the dilemma between virus infection and the host cell. There are many kinds of literature about EBV driving host cell cycle and promoting oncogenesis, but there are few kinds of literature on how the expression changes of host cell genes affected by EBV. EBV-encoded genes, such as LMP-1, EBNA-1, and EBNA-3C, have been shown to interact with E2F1 to affect cell growth; however, its concrete mechanism is not yet clear and needs further study. E2F1 is expected to be a new therapeutic target for EBV-associated malignancies.

EBV driving cell cycle and promoting oncogenesis is a very complex process. It is also necessary to further reveal its molecular regulatory network and key nodes to find more precise molecular targets and provide an effective solution for the prevention and treatment of EBV-related neoplasms.

## Abbreviations

EBV: Epstein–Barr virus
LMP: Latent membrane protein
EBNA: Epstein–Barr virus nuclear antigen
EBER: EBV-encoded small RNAs
